# Predicting Effects of the Self and Contextual Factors on Violence: A Comparison between School Students and Youth Offenders in Macau

**DOI:** 10.3390/ijerph15020258

**Published:** 2018-02-03

**Authors:** T. Wing Lo, Christopher H. K. Cheng

**Affiliations:** Department of Applied Social Sciences, City University of Hong Kong, Hong Kong, China; chris.cheng@cityu.edu.hk

**Keywords:** violence, Triad gangs, self-esteem, self-efficacy, family conflict, Macau

## Abstract

This study was designed to explore the self and contextual factors for violence in two samples of school students and youth offenders in Macau. There were 3085 participants who were between 12 and 20 years old; 48.3% of them were male and 51.7% female. Findings revealed that youth offenders exhibited more violence than school students. For the self factors, while lower self-esteem and higher self-efficacy of school students were associated with more violent behavior, these two variables had no significant effects for youth offenders. For the contextual factors, family conflict was the strongest predictor of violence, and school commitment/attachment was the weakest predictor for both samples. For youth offenders, family conflict had the largest direct effect, followed by susceptibility to negative peer influence and influence of the Triad gangs, while school commitment/attachment had a significant though mild direct effect. For school students, family conflict mediated the effect of self-esteem and self-efficacy on violence. While Triad gangs’ influence was the second strongest predictor of violence, being exposed to Triad gangs’ influence also mediated the effect of self-esteem and self-efficacy on violence. It is recommended that youth outreach services with a focus on family support and gang detachment for at-risk youth be strengthened.

## 1. Introduction

Macau, a former Portuguese colony and a casino city, was returned to China’s jurisdiction in 1999. In the mid-2000s, the Macau government opened its casino industry and allowed Western, Las Vegas-style entertainment business to invest in its gaming industry. Since then, with the influx of Chinese gamblers, Macau casinos have generated income to the government that exceeds that of Las Vegas by many times [1]. Macau has now achieved one of the highest volumes of GDP per capita in the world [2]. The sudden affluence has changed the social lives of the people in different areas. First, the affluence has rapidly increased the cost of living, especially rental and property prices, for the general public. In many families, both parents have to work full time and lack the time, energy, and patience to take care of their children. Family conflict has increased as a result of economic pressure in the family [3]. Moreover, because of the boom in the gaming industry and the construction of many new casinos and entertainment facilities, a social phenomenon of “low qualifications, high pay jobs” has emerged [3]. Because of its attractive salary and decent working environment, employment in the casinos has become a dream job for many young people, who in the past mainly relied on good academic qualifications for upward social mobility. However, those days are gone. Third, Macau was troubled by violence in the late 1990s because of the domination of Chinese Triad gangs in the operations of VIP rooms in casinos [4]. Despite a revamp of the casino industry, research suggests that the Triads maintain such domination. They treat the VIP rooms as their economic territories, and different kinds of betting and crime have emerged to cater to gamblers’ desires, thus creating a Triad-enterprise hybrid that exercises violence or has a reputation for violence in controlling a certain territory for financial gain [4,5,6,7]. To protect their business, the Triads recruit young people as junior gang members through a Dai Lo–Lan Tsai (protector–protégé) relationship to reinforce their territorial power [8]. People accept this behavior because they understand that the Triads’ reputation for violence represents a potential threat, and the Triads are capable of exercising force whenever they want [7].

Due to the increased economic pressure and conflict for families, continued Triad domination, and the “low qualifications, high pay jobs” phenomenon in the new gaming era, Macau has faced a problem of delinquency similar to that experienced by many affluent societies, and its government launched an initiative called Community Youth Work Teams in 2004 to assist at-risk youths [3]. However, published research on juvenile delinquency in Macau, let alone youth violence, has been rare. The present paper presents findings of the first study of youth violence in Macau. Its objectives are twofold. First, it aims to examine the etiology of youth violence from both self factors (self-esteem and self-efficacy) and contextual factors (family conflict, school attachment, peer influence, and gang participation), as revealed in the Western literature. Second, it will compare the violence exhibited by high school students and youth offenders and explore whether there are differences in the perceived self factors and contextual factors. The findings will reveal whether youth violence in Macau has unique causations or is not dissimilar to what has already been discovered in Western affluent societies. 

### 1.1. Self Factors: Self-Esteem and Self-Efficacy 

Research has found positive correlations between high self-esteem, high self-efficacy, and assertive behavior that include self-discipline and good work performance [9,10]. People having positive self-esteem and self-efficacy tend to self-accept, self-integrate, and self-achieve [11]. Youth develop positive self-concepts through recognition by family, teachers, and peers so that they have self-efficacy to face life challenges assertively and develop confidence in maintaining positive social relationships.

When youths strive for independence from family, they face numerous challenges in building self-identity. During this stage, rebellious behavior is a signal of youths’ search for growth and independence. If the family and school systems cannot give them sufficient support and assurance, appropriate social values and self-concepts may not be developed. Worse, a sense of low self-esteem may develop. Some youths may seek recognition from deviant peers to enhance their self-esteem. There is evidence supporting a correlation between negative self-esteem and delinquency [12]. It has been discovered that boys with low self-esteem could enhance self-esteem after engaging in delinquency; however, such an effect did not occur in boys with high self-esteem [13]. Although it has been reported that girls have less delinquent behavior than boys, girls with low levels of self-esteem have been associated with reported bullying behavior [14]. Comparatively high levels of reported engagement in peer bullying and relatively low self-esteem were each independently and significantly associated with the measure of delinquent behavior in both boys and girls [14]. 

Victimization and bullying also have been found to be negatively correlated with overall self-efficacy. In particular, the former has been shown to be negatively correlated with the traits of emotional intelligence and affective and cognitive empathy, whereas the latter was negatively correlated with the traits of emotional intelligence, empathy, and its cognitive component [15]. Aggressive youth have been shown to be more likely to hold violent attitudes and less likely to believe that conflict resolution could be achieved without violence [16]. Research suggests that reduced emotional self-efficacy is significantly associated with being threatened or injured with a weapon at school or bringing a weapon to school [17], and that perceived self-regulatory efficacy contributed to violent conduct both concurrently and longitudinally after controlling for prior level of violent conduct and openness of parental communication [18]. Psychopathic traits were found to be related to delinquent behavior in high risk youth and general youth population [19,20,21] Significant associations were found between youth psychopathic traits and conduct disorder, drug use, alcohol use, age of criminal onset, crime seriousness, and violent crimes [20].

### 1.2. Contextual Factors: Family, School, Peers, and Gangs

It has been found that both salient protective factors and risk factors for the development of violence and delinquency are rooted in the family system and are related to both positive and negative parenting behaviors [22]. An integrated and harmonious family system can provide children with intimacy and psychological support, promote prosocial values and healthy social development, and counteract the risk factors for juvenile delinquency [23]. In contrast, broken homes or permanently disrupted family structure has a consistent and reliable association with juvenile delinquency [24]. Events that accompany the dissolution of the family, rather than the event of divorce per se, have been suggested to be more salient predictors of childhood maladjustment. Cogent studies exploring different dimensions of the family process have substantiated these findings. The dimensions include the effects of family warmth and support, attachment and involvement, parental practices, parenting styles, and family conflict [23,24,25,26,27,28]. It has been discovered that positive parenting is a protective factor in which more positive parental coping strategies are associated with less relational aggression. In contrast, increased relational aggression has been shown to be associated with more harsh and uninvolved parenting. In these studies, interestingly, paternal but not maternal psychologically controlling parenting was positively related to relational aggression [22,29,30,31]. Similarly, another study found that only attachment to the father, and not to the mother, was predictive of youth aggressiveness [32].

A large body of research has shown that students who perform poorly in school and have low bonding and attachment to school are more likely to engage in serious forms of delinquency and violence [33]. Students who develop positive social bonds with their schools are more likely to perform well academically, and academic competence has often been used as an indicator of academic commitment [34]. Positive emotional and affective responses and participation in school-related activities have been found to have a protective effect on delinquency and violence [35]. Consistent discipline in parenting, a positive attitude to school, and prosocial behavior skills have buffering protective effects in school bullying [36]. In contrast, poor academic performance predicts not only the prevalence and onset of delinquency but also an escalation in its frequency and seriousness [37]. The effect between poor school bonding and delinquency is bidirectional: poor school bonding has been shown to predict delinquency over time, while delinquency also has been shown to predict poor school bonding [38]. Moreover, bullying perpetration at school is also a long-term predictor of aggression and violence [36]. 

Peer groups play an important role in adolescent socialization, and the significance of negative peer influence and gang delinquency on antisocial behavior and violence has been well documented [39,40,41,42]. While a school’s climate of safety has been shown to predict levels of gang participation by students, students’ perceptions of personal safety at school predicted their participation in gang activities [43]. Gang membership has been shown to be significantly associated with students’ academic performance, level of ethnic marginalization, drug availability in the community [44], and an increase in the odds of involvement in violence [45]. Research has found that having friends in gangs and low social self-control are positively associated with aggression, especially among boys, older and less educated youth [21,46,47]. Apart from being exposed to more deviant peers, youth gang members have also reported more family legal problems, suspension from school, attempts to run away from home, usage of alcohol and drugs, attempted suicide, street victimization, and parent–child conflicts, compared to non-gang youth [25,26]. Research suggests, however, that while membership in gangs is partially mediated by peer violence in its relation to individual violence, parenting practice is fully mediated in its relation to peer violence by gang membership [48].

## 2. Materials and Methods

### 2.1. Sampling

The present study involves a sample of 3085 young people recruited from high schools and juvenile homes in 2012. After ethical review was approved by the Research Ethics Committee of City University of Hong Kong (9231042), data collection from school students was facilitated by the Social Work Bureau of Macau using a multistage stratified random sampling approach. First, we divided Macau into eight geographic clusters; two to three secondary schools were randomly selected in each cluster, taking into account the student population size in the cluster. Among the sampled schools, stratified random sampling was used to select the classes of participants, giving an even distribution of students from each form (grade), ranging from 14.2% to 18.6%. As a result, 2755 students from 12 secondary schools were invited to take part in the study. The questionnaire was self-administered and anonymous; the data collection was conducted by two research assistants in the classroom without the presence of teachers. Besides school students, we also collected data from young people who were in conflict with the law. They had been sentenced by the court to reside in a juvenile and probation home or be supervised by a social worker in the community because they had committed an offense. Due to the nature of the participants, purposive sampling instead of stratified random sampling was used. With the assistance of the Social Work Bureau of Macau, 424 youth offenders were invited to participate in the study. The questionnaire was self-administered and anonymous; the data collection was conducted by a research assistant without the presence of agency officers or social workers. Altogether, 2755 school students and 330 youth offenders participated in the study (the rest refused to participate or their parents did not give consent), representing approximately 5.6% of the total population of young people between 12 and 20 years old in Macau.

### 2.2. Instruments

The questionnaire included measurement of the self (self-esteem and self-efficacy) and contextual variables (susceptibility to negative peer influence, attachment/commitment to school, Triad gang’s influence, and family conflict) that were hypothesized to predict the outcome variable (violence). It also included a section on demographic variables including age, gender, religion, education level, and place of birth. The Chinese language was used; measurement scales that were originally in English were translated into Chinese using a back-translation procedure.

#### 2.2.1. Self-Esteem 

The global self-esteem scale consisted of eight items adopted from the General Self subscale of the Chinese Adolescent Self-Esteem Scales (CASES) [49]. This instrument has demonstrated strong psychometric properties in terms of reliability and construct validity and has been widely used in Hong Kong for the assessment of young people’s self-esteem [50]. The response format adopted a 4-point Likert scale from 1 (strongly disagree) to 4 (strongly agree). The Cronbach’s α of this scale was 0.81, suggesting high internal consistency reliability. 

#### 2.2.2. Self-Efficacy 

The Chinese version of the self-efficacy scale was adopted from Schwarzer and associates [51]. It has 10 items designed to measure a person’s self-efficacy at the level of a general personality disposition. The items were mixed and randomized with the self-esteem items and followed the same 4-point Likert scale. The reliability coefficient Cronbach’s α was 0.76 in the current study.

#### 2.2.3. Susceptibility to Negative Peer Influence

Based on the Susceptibility to Peer Pressure Scale [52], three items that were deemed culturally valid were adopted. They were: “If your friend dares you to smoke, would you smoke?”; “If your friends are going to the movies and you have to study for a test, would you go with them?”; and “If your best friend skips school, would you skip too?” Two filler items about positive influence were constructed and added to this section for consideration of response set bias. Participants were to rate each item on a 4-point Likert scale, from 1 (absolutely no) to 4 (absolutely yes). The Cronbach’s α of this scale in the present study was 0.702. 

#### 2.2.4. Attachment and Commitment to School 

Thirteen items from Simcha-Fagan and Schwartz [53] were used to measure school attachment and commitment on a 4-point Likert scale, from 1 (strongly agree) to 4 (strongly disagree) as answers to statements such as “I feel picked on at school” or “I am bored at school”. The Cronbach’s α was 0.766.

#### 2.2.5. Triad Gang’s Influence 

The impact of Triad gang’s influence on the respondent was assessed on a 5-point Likert scale consisting of three self-constructed items: frequency of contact with Triad gang members, number of friends participating in Triad gangs, and the influence of Triad gang members in the school environment. These items were embedded randomly in different sections. The Cronbach’s α was 0.706.

#### 2.2.6. Family Conflict

A three-item scale measuring the degree of family conflict was constructed for the study. Questions were these: “In the last three months, how regularly have you quarreled with your family?”; “In the last three months, how regularly have you been physically assaulted by a member of your family?”; and “In the last three months, how regularly have you been shouted at by a member of your family?”. Responses were measured on a 5-point Likert scale, from 1 (never) to 5 (always). The Cronbach’s α was 0.721. 

#### 2.2.7. Violence 

Five questions about violent behaviors were asked, of which two items were constructed by the authors for the present study, and three items were adopted from Baldry and Farrington [54]. Examples of such behaviors included destroying or breaking windows of houses or shops and bullying, insulting, or fighting unknown people in the streets; the full list of items can be found in the Appendix. Other items of neutral or positive behaviors (e.g., browsing the Internet, shopping or window shopping, etc.) were added as filler items to minimize potential response set bias. Respondents were asked “In the last three months, how often have you…?” and responded on a 5-point Likert scale, from 1 (never) to 5 (always). The Cronbach’s α was 0.65. 

### 2.3. Data Analysis

**Figure 1:**
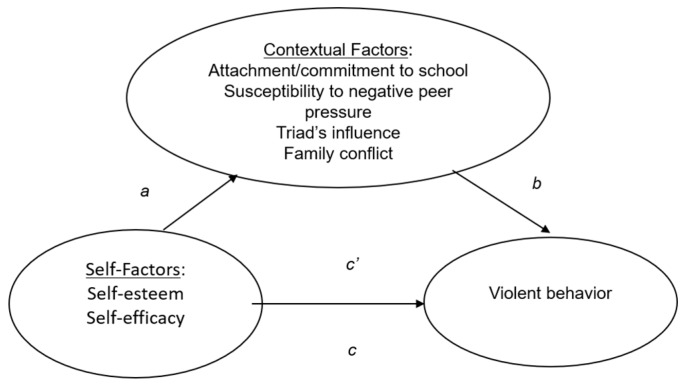
Conceptual path model of violence. Path *c* and *c’* indicates total and direct effects, respectively, whereas paths *a* × *b* indicates the indirect effect.

Statistical analyses were conducted using IBM SPSS version 23 (SPSS Inc., Chicago, IL, USA). Descriptive statistics of the variables and psychometric properties of the measurement scales were checked to ensure acceptable reliability and validity. For analysis of differences in violent behaviors reported by the school students and youth offenders, *t*-tests and multivariate analysis of variance (MANOVA) were conducted on the summative violence score (for *t*-tests) and each of the violent behaviors (for MANOVA). Hierarchical regression analyses were conducted to test the predictive effects of the self and contextual variables. Demographic variables including age, gender, place of birth, and religion were entered in the first step as controls, and other predictors (self-esteem, self-efficacy, susceptibility to negative peer influence, attachment/commitment to schools, Triad gang’s influence, and family conflict) were entered in subsequent steps to check for the additional variance explained. To analyze the mediating effects, the widely used approach suggested by Baron and Kenny [55] was employed. The conceptual path model of the mediating analysis is displayed in Figure 1. As a precondition for considering mediating effects, the paths *a, b,* and *c* must be statistically significant. Should there be mediating effects, the direct effect (path *c’*) of the independent variable on the dependent variables shall be reduced significantly when the mediating variables are controlled. The indirect effect of independent variable (IV) on dependent variable (DV) through mediating variable (MV) shall be indicated by the function of *a*∙*b*, and its significance is then checked by the Sobel test and the confidence intervals (CI) under bootstrapping. The Process macro [56] was used to conduct the mediating analyses. 

## 3. Results

### 3.1. Descriptive Statistics of the Samples

There were 3085 valid respondents in total, including 2755 school students and 330 youth offenders (representing response rates of 89.9% and 78%, respectively); 48.3% were male and 51.7% female (see Table 1). The mean age was 15.9 years (SD = 2.03). More than half of the respondents had received education up to the junior form level (55%), while more than one-third were educated to the senior form level (43.2%). A large majority of the respondents (78%) were born in Macau, while about 19% were from Mainland China, and the rest (2.5%) were from Hong Kong, Taiwan, or elsewhere. Regarding the youths’ parents, many had been educated to the secondary level (father: 29.0%; mother: 34.9%), around one-fifth were educated to the primary level (father: 20.9%; mother: 20.7%), nearly one-fourth had matriculated (father: 24.7%; mother: 23.1%) and only one-tenth or less had studied at tertiary institutions (father: 10.4%; mother: 8.7%). With regard to employment status, the modal category for fathers was semiskilled worker (29.6%) and for mothers was nonskilled worker (57.4%). The next largest group also differs by gender: 18.8% skilled workers for the fathers and 11.9% semiskilled workers for the mothers. Roughly 80% of the youths indicated that their parents were married. More than 40% of the youths did not know their monthly household income. Only one-fourth reported an income of less than MOP$15,000, with 18.4% falling into the category of MOP$15,000–$24,999, and 13.9% had an income greater than MOP$25,000 per month (US$1 = MOP$8, approximately). 

### 3.2. Psychometric Properties of Measurement Scales and Inter-Variable Correlations

Descriptive statistics and reliability coefficients of all measurement scales and the correlations between them are displayed in Table 2. All scales had acceptable to good internal consistency reliability (Cronbach’s alpha range from 0.65 to 0.81). Measurement validity was evaluated by inter-factor correlations. Convergent validity was supported by the positive correlations between self-esteem, self-efficacy, and attachment/commitment to school, and the positive correlation between violence, susceptibility to peer pressure, and Triad influence, whereas discriminant validity was shown by the negative correlations between family conflict, violence, and self-esteem. Overall, the pattern of inter-scale correlations suggested good construct validity of the measurement scales. 

### 3.3. Comparing the Prevalence of Violent Behavior between School Students and Youth Offenders

The two cohorts of young people showed very different prevalence in all five violent behaviors. Both an independent *t*-test and MANOVA were employed to test the differences. A summative violence score was computed for the *t*-test, while multiple scores on the violence items were entered for MANOVA (Table 3). As predicted, youth offenders exhibited more violence as a whole (*t* = 30.47, *p* < 0.001) than the school students did, as well as more of each kind of violent behavior (Pillai’s trace = 0.406, multivariate F_(5, 3042)_ = 415.70, *p* < 0.001). Of the five violent behaviors, *physical bullying* had the highest prevalence in youth offenders but the lowest prevalence among the school students. The largest effect size indicating the difference between the two cohorts of young people was also found for *physical bullying*, F_(1, 3046)_ = 1935.42, *p* < 0.001, η^2^ = 0.39. For school students, *vandalizing walls in public areas* was of highest prevalence among different violent behaviors. The second largest difference between the two cohorts was in the prevalence of *destroying or breaking windows of other people’s houses*, F_(1, 3046)_ = 790.94, *p* < 0.001, η^2^ = 0.21.

### 3.4. Multiple Regression Analysis and Mediation Analysis

Hierarchical regression analysis was conducted to evaluate the predictive effects of the self and contextual variables on violence. In a first step, demographic variables (age, gender, place of birth, and religion) were entered as controls, and total score in violence was the outcome (predicted) variable. Self-esteem and self-efficacy were entered in the second step, followed by contextual variables (susceptibility to peers’ influence, attachment/commitment to school, Triad gang’s influence, and family conflict) in the last step. Some demographic variables (age and gender) had an association with violence for school students but not for youth offenders (see Table 4). For school students, students who were older (β = 0.15, *p* < 0.001) and male (β = −0.16, *p* < 0.001) tended to have more violent behaviors than younger and female students; religion and place of birth did not have any relationship with violence. For youth offenders, none of the demographic variables had any effect on violence. For school students, self-esteem (β = −0.20, *p* < 0.001) and self-efficacy (β = 0.07, *p* < 0.01) had mild but significant effects on violence, suggesting that lower self-esteem and higher self-efficacy of school students were associated with more violent behavior. However, the self-variables did not have significant effects for youth offenders (*p* > 0.05). For both school students and youth offenders, the contextual variables had added the explained variance (on violence) significantly, *R^2^ change* = 0.56 and 0.73, respectively, for school students and youth offenders. Among the four contextual variables, *family conflict* had the strongest predictive effect on violence for both school students and youth offenders (β = 0.62 and 0.54, respectively). For youth offenders, *susceptibility to negative peer influence* (β = 0.28) and *Triad’s influence* (β = 0.25) had the second and third largest effects on violence. However, for school students, *susceptibility to negative peer influence* was in the third place (β = 0.14) among the four contextual variables. *School commitment and attachment* had the lowest effect, although a still significant one, on violence for both youth offenders and for students (Table 4). To summarize, *family conflict* was most predictive of young people’s violent behavior, while *school attachment or commitment* had the lowest though still significant association with violence. The self factors (self-esteem and self-efficacy) of youth offenders did not seem to have any relationship with their violent behavior, but the self factors of school students had significant though mild effects on their violent behavior.

Mediating analyses were conducted using the Process macro [56]. The contextual factors were entered as mediating variables (MV) that would mediate the effect of self-esteem or self-efficacy on violence; demographic variables were controlled as covariates. The analyses were conducted on school students and youth offenders separately. It was found that for school students, self-esteem had significant negative effects on violence, and all contextual factors had direct effects on violence and also significantly mediated the relationship between self-esteem and violence (see Table 5). Total effect and all indirect effects of self-esteem (through the four contextual factors) were negative, meaning that lower self-esteem was predictive of more violence, and this association could be mediated through stronger influence of peers and Triad gangs, more family conflicts, and lower attachment or commitment to schools. Among the four contextual factors, *family conflict* and *Triad’s influence* had the strongest direct effects and mediating effects. 

However, the results found for the youth offenders were quite different (Table 6). For youth offenders, self-esteem had no significant effect on violence, either as a total, direct, or indirect effect. That is to say, for youth offenders, their self-esteem was not significantly predictive of violence nor was it mediated by the contextual factors. Instead, the four contextual factors had significant direct effects on their violent behavior. Among them, *family conflict* had the largest direct effect (*t* = 15.53, *p* < 0.0001), followed by *susceptibility to negative peer influence* (*t* = 7.82, *p* < 0.0001) and *Triad gang’s influence* (*t* =7.10, *p* < 0.0001), while *school commitment/attachment* had a significant though mild direct effect (Table 6). 

As for the role of self-efficacy, it was found that only school students’ self-efficacy had significant effects (both direct and indirect) on their violent behavior, but it did not have any effect on violence among the youth offenders (Table 7 and Table 8). Actually, self-efficacy did not have any effect (total, direct, or indirect) on violence for the youth offenders. It was interesting to note that, for school students, the relationship between their self-efficacy and violence was a positive one, meaning that higher self-efficacy was associated with more violence, which was unlike the negative association between self-esteem and violence. Furthermore, self-efficacy had indirect effects on violence through some contextual variables in the school students sample; the indirect effects were mediated through *family conflict* (Sobel’s Z = −6.69, *p* < 0.0001), *school attachment/commitment* (Sobel’s Z = 2.01, *p* < 0.001), and *Triad gang’s influence* (Sobel Z = 2.01, *p* < 0.05). However, such indirect effects were not found in the youth offenders (all indirect effects of self-efficacy were nonsignificant).

To check whether the predictor and mediating variables could reversely account for violence, we reversed the IVs and MVs in the mediating analysis model. Results showed that contextual variables had much stronger direct effects than self-variables, which had no significant mediating effect on the relationship between contextual factors and violence. Hence, evidence for the predictive mediating roles of self-esteem, self-efficacy, and the four contextual factors could be confirmed. 

## 4. Discussion

In filling gaps of knowledge on youth violence in Asia’s highest income-generating casino city, the present study found self-reported rates of youth violence with multiple causes and effects similar to Western affluent societies. The findings revealed that youth offenders committed more violence than school students. Similar to previous studies where the influence of age and gender was more robust [19,47], male and older students committed more violence than female and younger students. Violence was caused by both self and contextual factors. All four contextual factors used in this study—family conflict, Triad gang association, negative peer influence, and school unattachment—were associated with violence in both students’ and youth offenders’ groups, but family conflict was found to be the strongest predictor of youth violence. The effect of several mediators was discussed. Family conflict had the strongest direct effect on violence, and it also mediated the effect of self-esteem and self-efficacy on violence for school students. 

Familial conflict has been found in numerous studies to be associated with delinquency, crime, and violence, and there is evidence to even suggest that parental conflict is the most salient predictor of children’s maladjustment [25]. When compared with other groups of obstetric and poverty risk factors, children with both early neuromotor deficits and unstable family environments have been shown to have significantly more academic and behavioral problems in adolescence and even double the rates of violence, theft, and total crime in adulthood [57]. Although genetic and epigenetic processes may contribute to family-based risks before birth, family conflict and disrupted parenting, such as exposure to violence and harshness, may impact the development of children [23]. For parents, patriarchal beliefs, substance abuse, mental illness, and a history of child abuse, as well as parental trauma experiences and stressful parent–child interaction, were found to be risk factors [29]. Thus, findings of the present study on the relationship between family conflict and violence in Macau are consistent with the Western literature and are also in line with previous Macau studies that the rapidly changing economic structure has gradually induced more family disharmony [3].

School is described as an eminently conventional institution, as positive bonding with the school can buffer the students against violence. Students who bond with their schools are less likely to become minor or serious offenders. They spend a large proportion of their time in school, making schools important institutions for socializing. Weakened bonds with the school have been consistently found to be a major source of delinquency [58]. However, in the present study, although there is a significant relationship between school commitment and violence in both the students’ and youth offenders’ groups, the relationships are weak. This can be interpreted by the changing social reality in Macau, i.e., its gaming industry requires a large pool of young people to staff its operations, but it looks for street smarts rather than book smarts [10]. Tertiary education is not highly valued by young people and their families [3] because casinos provide many “low qualification, high pay jobs” that do not require youth with high academic qualifications.

Western research has found that underperforming school students are likely to group together for mutual support [59]. Because of social labeling, they are resigned to perform badly to be accepted in their delinquent peer group or form their own gang subculture [42,59,60]. Gang attachment and delinquent peer association increase the likelihood of youth crime and violence [61]. In the present study, Triad gang association and negative peer influences were found to be the second and third strongest predictor of violence for both school students and youth offenders. There was a very strong and significant correlation (0.57, ** *p* < 0.001) between Triad gang participation and negative peer influence (see Table 2). If youths fail to gain acceptance and satisfaction through the family and school systems, impaired self-concepts would emerge. Research in Chinese communities has found that some youths with low self-esteem may become victims of violence [62,63]. Some others may attempt to gain social recognition from delinquent peers and gang members through bullying or violence on other victims [59,64,65,66].

Our findings suggested that lower self-esteem and higher self-efficacy of school students were associated with more violent behavior, but the relationships were weak. It has been acknowledged that self-esteem and self-efficacy have equal weight in contributing to delinquency and violence [67,68]. Deviant behavior may be an adaptation and self-protection against self-derogation. If a sense of self-esteem and positive self-evaluation cannot be gained through socially acceptable methods, motivation for behaving in positive ways will gradually decline. Instead, one may try to gain self-esteem and attention through other means that may include delinquency and violence [69]. Thus, having low self-concepts has often been regarded as a motivation for violence. However, in this study, it was interesting to note that, for school students, the relationship between their self-efficacy and violence was a positive one, revealing that higher self-efficacy was associated with more violence, which was unlike the negative association between self-esteem and violence. This suggests that students with high self-efficacy had the courage to exert violence, or after they committed violence, their self-efficacy was enhanced (further discussed below).

However, in the youth offenders’ group, we found no significant relationship between self factors and violence, but there was a relationship between negative peer influence and Triad gang participation and violence. Research suggests that it is sometimes not the violence itself, but rather the acceptance and recognition gained from deviant peers and gang members after committing violence, that enhances the youth’s self-esteem [65,66,70]. A youth offender’s sense of self-enhancement and protection can ultimately be gained by strengthening his or her psychological bonds with other delinquent peers when they engage in similar behavior. Therefore, there is not necessarily a direct relationship between self-esteem, self-efficacy, and violence, which can be enhanced through delinquent peer association, support, and recognition [65,66,71]. 

Many youth offenders in this study were already Triad gang members. Once a youth is inside a gang, violence is regarded as an expression of conformed behavior within the gang, where members’ acceptance of antisocial behavior becomes a reward for committing violence [8,65]. The enhancement of self-esteem through other members’ recognition is a significant factor in their gang participation. In the present study, for school students (but not for youth offenders), being exposed to Triad gangs’ influence also mediated the effect of self-esteem and self-efficacy on violence. In addition, while higher self-efficacy was associated with violence in the students’ group, it was not in the youth offenders’ group. The findings suggest that a developmental process may have emerged. Initially, the school students’ self-esteem is relatively low and they have a strong desire to be accepted by peers, and committing violence is one option. Once they begin to commit violent behavior, their self-efficacy is gradually enhanced, and the enhanced self-efficacy would facilitate further violence, until they have established status within their peer groups [65,66]. Some may even join Triad gangs, commit crime, and have encounters with the police. Eventually, when they become role models for other deviant students, their self-concept is strengthened. At this later stage, violence is no longer affected by their low self-concept, but by gang participation. Once they belong to a gang, the gang subculture would guide their violent behavior. Within the subculture, the youths commit violence to fulfil the gang norms of “eye for an eye” and Triad brotherhood [72]. The reward and punishment mechanisms within the gang reinforce members to conform to the violent and brotherhood norms [8].

## 5. Conclusions 

Adolescence is a restless and stormy period, and young people at this stage are striving for the development of positive self-concepts, a sense of independence, and the learning of proper social norms so that they can be accepted and trusted by others, particularly their peers [39]. Family and school can be two-edged swords: they are places for the youths’ psychological and social growth, but disrupted family life and school failures may also contribute to delinquency and violence. For youth who suffer from family and school problems, or who have low self-concepts, delinquent peers and gangs are ready to accept their antisocial behavior and provide them with support and recognition [45,65,66]. However, one limitation of the present study is that it cannot identify whether the deviant school students seek out and join the gangs themselves, or whether the gangs recruit them because of their violent potential.

Intervention and prevention programs should target the modification of youths’ violent attitudes through early identification of students with low self-esteem and control, and enhancement of self-efficacy in problem-solving without resorting to aggression. Such programs should strengthen the youths’ coping skills that help them to resist negative peer influence, and support the development of a harmonious family environment and positive parenting styles [16,29,31,46,73]. To change the negative self-image, rebuilding of positive self-identity would prevent them from going astray. Since Macau has already established Community Youth Work Teams whose social workers outreach to different neighborhoods to help at-risk youths [3], the teams should continue the services that aim to prevent youth from participating in gang activities or shorten their gang life through social and emotional learning and violence prevention programs [17,42,45,74]. Further studies need to be conducted to assess the effectiveness of different services provided to at-risk youth, and different working approaches adopted for helping these individuals, during a time of rapid social change when the gaming industry ignites the disruptions in the economic structure, thus undermining family cohesion and narrowing the career path of young people in Macau.

## Figures and Tables

**Table 1:** Sample characteristics (%).

Variable	School Students (*N* = 2755)	Youth Offenders (*N* = 330)	Total (*N* = 3085)
Gender			
Male	46.0	68.2	48.3
Female	54.0	31.8	51.7
Age; M (SD)	15.86 (2.07)	16.20 (1.58)	15.90 (2.03)
12	8.5	1.2	7.8
13	12.1	4.3	11.2
14	14.9	14.7	14.8
15	16.6	25.4	17.5
16	16.5	22.1	17.0
17	14.8	19.2	15.2
18 to 20	16.6	12.6	15.0
Education			
Primary and below	0	19.7	2.1
Junior (forms 1–3)	52.8	72.4	54.9
Senior (forms 4–6)	47.2	8.0	43.0
Monthly Household Income (USD$1 = MOP$8)			
Below MOP$15,000	42.1	50.9	43.0
MOP$15,000–24,999	33.9	19,4	32.5
MOP$25,000 and above	24.0	29.7	24.5
Father’s Education Level			
Primary and below	20.4	25.3	20.9
Junior (forms 1–3)	29.1	28.1	29.0
Senior (forms 4–6)	25.8	15.3	24.7
Tertiary	10.9	6.6	10.4
Do not know	13.8	24.7	15.0
Mother’s Education Level			
Primary and below	20.3	23.6	20.7
Junior (forms 1–3)	35.4	31.1	34.9
Senior (forms 4–6)	23.5	19.9	23.1
Tertiary	9.2	5.0	8.7
Do not know	11.7	20.5	12.6
Father’s Employment Status			
Manager level and professional	13.1	9.0	12.6
Skilled worker and business owner	19.4	13.8	18.8
Semi-skilled worker	29.6	28.9	29.6
Non-skilled worker	17.2	17.9	17.3
Unemployed	2.6	5.4	2.9
Retired	1.7	1.0	1.6
Others	5.8	4.8	5.7
Do not know	10.0	18.9	11.0
Mother’s Employment Status			
Manager level and professional	10.6	7.9	10.4
Skilled worker and business owner	7.6	5.4	7.4
Semi-skilled worker	12.4	8.2	11.9
Non-skilled worker	57.5	58.2	57.4
Unemployed	0.8	1.9	0.9
Retired	0.3	1.3	0.4
Others	4.5	5.1	4.5
Do not know	7.3	12.3	7.8
Parents’ Marital Status			
Married	81.1	62.7	79.2
Cohabiting	6.1	5.5	6.1
Separated and divorced	10.0	27.6	11.9
Widowed	2.4	4.2	2.6

**Table 2:** Descriptive statistics, internal consistency reliability, and inter-variable correlations of all measurement scales.

Caption	Self-Esteem	Self-Efficacy	Peers	School	Triad	Family	Violence	Cronbach’s *α*
*Self-esteem*	1.0							0.81 (8 items)
*Self-efficacy*	0.58 **	1.0						0.76 (10 items)
*Peers*	−0.07 **	0.05 **	1.0					0.70 (3 items)
*School*	0.26 **	0.13 **	−0.52 **	1.0				0.77 (13 items)
*Triad*	−0.05	0.07 *	0.57 **	−0.40 **	1.0			0.71 (3 items)
*Family*	−0.22 **	−0.10 **	0.29 **	−0.29 **	0.31 **	1.0		0.72 (3 items)
*Violence*	−0.11 **	0.03	0.59 **	−0.44 **	0.64 **	0.69 **	1.0	0.65 (5 items)
Mean	2.87	2.66	1.72	2.95	0.376	0.943	3.10	
SD	0.52	0.39	0.58	0.39	0.69	0.851	3.06	

Note: Peers, Susceptibility to negative peer pressure; School, Attachment /commitment to school; Triad, Triad’s influence; Family, Family conflict. Self-esteem, Self-efficacy, Peers, and School were coded in a four-point scale from 1 (Strongly Disagree /Surely Not) to 4 (Strongly Agree / Surely Yes); Triad and Family were coded in a five-point scale from 0 (Never) to 4 (Always); * *p* < 0.01, ** *p* < 0.001.

**Table 3:** Violent behaviors of school students and youth offenders.

Behavior	School Students (*N* = 2723)		Youth Offenders (*N* = 325)		*F*-test	Effect Size
	M	(SD)	M	(SD)		η^2^
Vandalizing walls in public places	1.29	(1.09)	1.92	(1.34)	90.98 **	0.029
Bullying, insulting, fighting in the streets	0.31	(0.72)	0.83	(1.11)	132.44 **	0.042
Physically bullying others	0.12	(0.57)	2.05	(1.56)	1935.45 **	0.389
Carrying a weapon in case needed in a fight	0.40	(0.88)	0.69	(1.07)	30.59 **	0.010
Breaking windows or destroying other people’s houses or shops	0.46	(0.81)	1.90	(1.27)	790.94 **	0.0206

Note: *p < 0.01, ** *p* < 0.001; Multivariate tests: Pillai’s trace = 0.406, F_(3042, 5)_ = 415.7.

**Table 4:** Hierarchical regression analysis of violent behavior predicted by self and contextual variables.

Caption	Step 1 (β)		Step 2 (β)		Step 3 (β)	
Predictors	*Youth Offenders*	*School Students*	*Youth Offenders*	*School Students*	*Youth Offenders*	*School Students*
Age	0.04	0.15 **	0.04	0.15 **	0.07	0.12 **
Gender	0.07	−0.16 **	0.07	−0.16 **	0.02	−0.09 **
Birthplace	0.06	−0.04	0.06	−0.03	0.02	−0.02
Religion	0.08	0.04	0.09	0.04	0.03	0.02
Self-esteem			−0.14	−0.20 **	−0.06	0.04
Self-efficacy			0.10	0.07 *	0.10	0.02
Peers					0.28 **	0.14 **
School					−0.10 *	−0.04 *
Triad					0.25 **	0.25 **
Family					0.54 **	0.62 **
*R*	0.12	0.23 **	0.15	0.29 **	0.87 **	0.80 **
*R*^2^ change	−	−	0.01	0.03 **	0.73 **	0.56 **

Note: Predicted Variable: Violence; Peers = Susceptibility to negative peer pressure; School = Attachment /commitment to school; Triad = Triad’s influence; Family = Family conflict; *p < 0.01, ** *p* < 0.001.

**Table 5:** Mediating analysis of contextual factors on the relationship between self-esteem and violence of school students.

Effects	IV	MV	Coefficient	*t*-Test	*p*	95% CI ^	
Total (*c*)	Self-esteem		−0.73	−8.16	<0.0000		
Direct (*c’*)	Self-esteem		00.16	2.72	0.0066		
Direct (*b*)		Peers	0.66	9.27	<0.0000		
		School	−0.25	−2.77	0.0056		
		Triad	1.24	18.27	<0.0000		
		Family	1.83	48.10	<0.0000		
Indirect effect (*a.b* path)	Self-esteem (via MVs)		−0.89	−11.85 (Sobel Z)	<0.0000	−1.055	−0.702

Note. Predicted Variable: Violence; ^ CI based on 1000 bootstrapping samples.

**Table 6:** Mediating analysis of contextual factors on the relationship between self-esteem and violence of youth offenders.

Effects	IV	MV	Coefficient	*t*-Test	*p*	95% CI ^	
Total (*c*)	Self-esteem		−0.57	−1.17	0.242		
Direct (*c’*)	Self-esteem		0.10	0.40	0.687		
Direct (*b*)		Peers	1.80	7.82	<0.0000		
		School	−0.92	−2.65	0.0086		
		Triad	0.97	7.10	<0.0000		
		Family	2.14	15.53	<0.0000		
Indirect effect (*a.b* path)	Self-esteem (via MVs)		0.67	−1.91 (Sobel Z)	0.0557	−0.009	0.499

Note. Predicted Variable: Violence; ^ CI based on 1000 bootstrapping samples.

**Table 7:** Mediating analysis of contextual factors on the relationship between self-efficacy and violence of school students.

Effects	IV	MV	Coefficient		*t*-Test	*p*	95% CI ^
Total (*c*)	Self-efficacy		−0.07	−0.56	0.5741		
Direct (*c’*)	Self-efficacy		0.40	5.26	<0.0000		
Direct (*b*)		Peers	0.76	10.56	<0.0000		
		School	−0.37	−4.11	<0.0000		
		Triad	1.33	19.61	<0.0000		
		Family	1.81	47.72	<0.0000		
Indirect effect (*a.b* path)	Self-efficacy (via MVs)		−0.47	−4.79 (Sobel Z)	<0.0000	−0.759	−0.213

Note. Predicted Variable: Violence; ^ CI based on 1000 bootstrapping samples.

**Table 8:** Mediating analysis of contextual factors on the relationship between self-efficacy and violence of youth offenders.

Effects	IV	MV	Coefficient	*t*-Test	*p*	95% CI ^	
Total (*c*)	Self-efficacy		−0.27	−0.45	0.653		
Direct (*c’*)	Self-efficacy		0.55	1.77	0.078		
Direct (*b*)		Peers	1.79	7.99	<0.0000		
		School	−1.07	−3.12	0.0020		
		Triad	0.95	7.05	<0.0000		
		Family	2.11	15.71	<0.0000		
Indirect effect (*a.b* path)	Self-efficacy (via MVs)		−0.82	−1.58 (Sobel Z)	0.1149	−2.057	0.773

Note. Predicted Variable: Violence; ^ CI based on 1000 bootstrapping samples.

## Appendix A. Violent Behaviors

1. Bullying, insulting, fighting unknown people in the streets 2. Physically bullying others 3. Carrying a weapon in case needed in a fight 4. Vandalizing walls in public places 5. Destroying or breaking windows of houses or shops
